# Awareness of HIV-positive status and linkage to treatment prior to pregnancy in the “test and treat” era: A national antenatal sentinel survey, 2017, South Africa

**DOI:** 10.1371/journal.pone.0229874

**Published:** 2020-03-13

**Authors:** Selamawit Woldesenbet, Tendesayi Kufa, Mireille Cheyip, Kassahun Ayalew, Carl Lombard, Samuel Manda, Patrick Nadol, Peter Barron, Brian Chirombo, Ehi Igumbor, Yogan Pillay, Adrian Puren

**Affiliations:** 1 Center for HIV and STI, National Institute for Communicable Diseases, Johannesburg, South Africa; 2 School of Public Health, University of the Witwatersrand, Johannesburg, South Africa; 3 Strategic Information Unit, Center for Disease Control and Prevention, Pretoria, South Africa; 4 Biostatistics Unit, South African Medical Research Council, Cape Town, South Africa; 5 Biostatistics Unit, South African Medical Research Council, Pretoria, South Africa; 6 HIV and Hepatitis Program, World Health Organization, Pretoria, South Africa; 7 School of Public Health, University of the Western Cape, Bellville, Cape Town, South Africa; 8 HIV & AIDS, TB and Maternal, Child and Women’s Health (MCWH), National Department of Health, Pretoria, South Africa; 9 Virology Department, University of the Witwatersrand, Johannesburg, South Africa; Albert Einstein College of Medicine, UNITED STATES

## Abstract

**Introduction:**

Knowledge of HIV status in South Africa (SA) is reported to be 90% among people living with HIV. National level estimates could mask population-specific levels, which are critical to monitor program coverage and potential impact. Using data from the 2017 national antenatal sentinel survey, we assessed knowledge of HIV-positive status, initiation of antiretroviral therapy (ART), and socio-demographic characteristics associated with knowledge of HIV-positive status prior to the current pregnancy among women attending antenatal care.

**Methods:**

Between 1 October and 15 November 2017, a nationally representative sample of 32,716 pregnant women were enrolled from 1,595 public health facilities selected from all districts of SA. Data on age, gravidity, knowledge of HIV-positive status and ART initiation prior to pregnancy were extracted from medical records. A blood sample was collected from each woman regardless of prior knowledge of HIV status or ART history, and tested for HIV in the laboratory. All HIV-positive pregnant women enrolled in the survey were eligible for inclusion in the analysis. Multivariable survey logistic regression was used to examine factors associated with knowledge of HIV-positive status prior to the current pregnancy.

**Results:**

Of 10,065 eligible HIV-positive women, 60.8% (95% confidence interval (CI):59.9%–61.7%) knew their HIV status prior to the current pregnancy, of whom 91.1% (95% CI: 90.4%–91.7%) initiated ART prior to the current pregnancy. Knowledge of HIV-positive status was lower among adolescent girls and young women (15–24 years) (38.9%) and primigravid women (40.5%) compared with older women (35–49 years) (75.5%) and multigravid women (64.7%). In a multivariable analysis, significant effect modification was found between gravidity and age (P value = 0.047). Being in the age group 15–24 years compared to the age group 35–49 years decreased the odds of knowing HIV-positive status by 80% (adjusted odds ratio (AOR): 0.2, 95% CI:0.1–0.4) among primigravid women and by 60%(AOR: 0.4, 95% CI:0.3–0.4) among multigravid women.

**Conclusion:**

Knowledge of HIV-positive status prior to the current pregnancy fell short of the target of 90% among pregnant women living with HIV. This was especially low among adolescent girls and young women, highlighting the gap in youth friendly reproductive health and HIV testing services.

## Introduction

Early treatment with life-long antiretroviral therapy (ART) is a highly effective strategy to prevent both vertical and horizontal transmission of HIV as well as HIV-related morbidity and mortality [1, 2]. To accelerate the scale-up of ART, in 2014, the Joint United Nations Programme on HIV/AIDS (UNAIDS) launched the 90–90–90 and the 95–95–95 targets, which aim that, by 2020 and 2030, respectively, 90% and 95% of people living with HIV (PLHIV) know their HIV status, 90% and 95% of those who know their HIV-positive status receive ART, and 90% and 95% of those on ART have viral suppression [3].

Prevention of mother-to-child transmission (PMTCT) is a significant pathway and an effective HIV testing modality for diagnosing and linking women to ART. The first and the second 90 in the PMTCT programme has been reported to be >90% in South Africa (SA), but knowledge of HIV status and ART initiation prior to pregnancy has been reported to be low [4, 5]. In 2016, SA adopted the universal test and treat (UTT) strategy, which makes provision for ART initiation immediately after an HIV-positive test [6]. UTT will enable more HIV-positive women to access ART services prior to pregnancy and conceive with an undetectable viral load, which is critical for PMTCT as well as maternal health [7, 8]. Initiating treatment as early as possible before pregnancy or early in pregnancy enables to achieve undetectable viral load at delivery and reduces the risk of in-utero and intrapartum transmission [9].

Limited studies have assessed knowledge of HIV status and ART initiation prior to pregnancy in the UTT era and the demographic characteristics of women who access (or do not access) HIV testing and ART services prior to pregnancy. Improved understanding of the demographic profile of HIV-positive pregnant women who do not access ART services prior to pregnancy can help implement interventions appropriate for this population.

SA has been implementing the national antenatal HIV sentinel survey (ANCHSS) since 1990: annually until 2015, and biennially since 2015. Between 1990 and 2015, the survey primarily focused on estimating HIV prevalence trends over time, using anonymous unlinked testing of blood samples collected from pregnant women attending routine antenatal care (ANC). In the 2017 survey, with the introduction of the 90–90–90 targets, in order to monitor the impact of HIV prevention and treatment programmes, additional data have been gathered on knowledge of HIV-positive status (1st 90) and ART coverage (2nd 90).

Using data from the 2017 ANCHSS, this study assessed knowledge of HIV status prior to the current pregnancy, ART initiation prior to the current pregnancy and the socio-demographic characteristics associated with knowledge of HIV-positive status prior to the current pregnancy among pregnant women.

## Materials and methods

### Study design

This was a cross-sectional ANCHSS involving HIV testing of pregnant women aged 15–49 years attending ANC at public health care facilities throughout SA. Sites were selected using stratified multistage cluster sampling method. The sampling frame for the primary sampling unit (PSU) consisted of public health care facilities providing ANC services. Eligible sentinel sites were selected according to the Probability Proportional to Size (PPS) sampling method using annual volume of ANC clients as proxy for size of facility. A detailed discussion on sample size and sampling of sites is presented elsewhere[10].

Eligible facilities that took part in the 2015 survey were included in the 2017 survey. To be included as a sentinel surveillance site, the public health care facility had to provide pregnancy testing and ANC services (99% of primary health care facilities in South Africa provide ANC); have a minimum of 20 first-time ANC attendees per month(85% of public facilities in South Africa have a minimum of 20 first-time ANC attendees per month); draw blood routinely from first-time ANC attendees, with facilities to store specimens at 4 degrees Celsius (°C); and be ready to transport biological specimens to the nearest regional laboratory within 24 hours. The survey was limited to public health facilities. Sites were not selected based on local epidemiology or interventions.

### Sample size

The sample size was calculated for the primary objective of the study to measure HIV prevalence at district level within a precision of 3–5%. It was envisaged that 36,015 pregnant women from 1,595 public health facilities would be included: the number of sites selected per district ranged from 8 to 83. For this analysis, we only included HIV-positive pregnant women identified in the survey. With the sample size calculated for the primary objective, it was possible to estimate knowledge of HIV status and ART initiation prior to pregnancy among HIV-positive women within 1.3% precision at national level. This is calculated assuming knowledge of HIV-positive status and ART initiation of 60% and 55% respectively, HIV prevalence of 30%, with 95% confidence interval (CI), design effect of 1.5 and 10% error rate (to account for loss of specimen, breakage, and incomplete/missing data).

### Data collection

The survey was conducted between 1 October and 15 November 2017. Data were collected by health workers providing routine ANC service. Before the scheduled commencement date of the survey, a one-day training session on the aims of the survey and data collection procedures, was held using the training of trainers approach, at national level, in all nine Provincial Health offices and at district level. Further detail about the content of the training is provided in the main report[10].

During the designated enrolment period, each pregnant woman attending an ANC visit at a sentinel site was given the opportunity to enrol into the survey. Consecutively, consenting pregnant women aged 15–49 years who attended the antenatal clinic in the survey period, either for the first-time or for follow-up visits during their current pregnancy, were eligible, regardless of their HIV status or history of routine HIV tests.

The data collection procedures included obtaining *written* informed consent, a brief interview using a standardized data collection form, data abstraction from medical records, and blood specimen collection. Blood specimen was taken from each woman, regardless of prior knowledge of HIV status or ART history, and tested for HIV in the laboratory. Demographic and clinical information collected through interview included race of the woman, level of education, marital status, gravidity, and parity. Data on province, age of the woman, HIV status at first ANC visit or enrolment in the survey, knowledge of HIV-positive status prior to pregnancy, and ART initiation prior to pregnancy were extracted from medical records of enrolled women.

### Specimen testing for HIV

Standardized HIV testing procedures, as outlined in the national HIV testing guideline (2016), were used [11]. Two fourth-generation HIV-1 enzyme immunoassays (EIA) were used to test for HIV infection, following the manufacturer’s instructions–including appropriate quality control specimens. All plasma samples were tested at the regional laboratories, using the screening assay (EIA 1). Specimens that were non-reactive on EIA 1 were classified as HIV negative and were not tested further. All samples that were reactive on EIA 1 were further tested using the confirmatory assay (EIA 2). If specimens were reactive on EIA 2 they were classified as HIV-positive. If EIA 2 was non-reactive, the specimen was considered to have a “discrepant” HIV result.

The final HIV test results were returned to participants (through the clinic) if they were unaware of their HIV status or if there was a discrepancy between the results of the survey-provided laboratory test and the documented routine clinic test.

### Data management

Completed data collection forms were captured in District Health Information System (DHIS) by data clerks. All HIV screening (EIA 1), confirmatory (EIA 2) and final HIV test results were captured into the National Health Laboratory Services (NHLS) laboratory information management system (TrakCare) and exported to Microsoft Excel (2016 version). The laboratory data were then merged with the interview data captured on DHIS, using STATA® 14 (Stata Corporation.College Station, TX) for data cleaning and analysis.

The final database excluded observations for participants outside the age range of 15–49 years, those with no interview data, rejected or lost specimens and those with equivocal or unconfirmed (discrepant) HIV test results. In addition, for this analysis, all HIV negative participants (per laboratory test) and participants who had a missing response for the question “knowledge of HIV-positive status prior to the current pregnancy” were excluded as the aim of this study was to measure knowledge of HIV status (1^st^ 90) and ART initiation among HIV-positive participants only.

### Data analysis

Data analysis took into account the survey design (clustering within PSUs, and stratification by district) and was weighted for the Statistics SA (Stats SA) 2017 mid-year population size of women of reproductive age (15–49 years), at province level [12]. A population finite correction factor was added to adjust for the >5% of PSUs sampled without replacement from a finite population of about 4,000 public facilities.

Descriptive analyses included a summary of sample size realization and data distribution by province, nationally and by demographic characteristics. Median and interquartile ranges (IQR) were reported for continuous variables, while frequencies were reported for categorical variables. The primary outcomes for this analysis were knowledge of HIV-positive status prior to the current pregnancy (1st 90) and ART initiation prior to the current pregnancy (2nd 90). The denominator for knowledge of HIV-positive status prior to the current pregnancy (1st 90) was the number of HIV-positive pregnant women (per EIA test). The denominator for ART initiation prior to the current pregnancy (2nd 90) was the number of HIV-positive pregnant women who knew their HIV-positive status prior to the current pregnancy. The 1st 90 and the 2nd 90 were compared by demographic characteristics and province. *P* values from chi-square tests are reported for such comparisons.

A survey domain based multivariable logistic regression was used to examine factors associated with knowing HIV-positive status prior to first ANC visit [13]. Variables significant at *P* value cut-off point of 0.2 in a bi-variable model and variables known a priori to be strongly associated with the outcome variable were included in a multivariable model. From a multivariable model, variables were removed from the model if they were not statistically significant and do not affect the coefficients of other variables by 10% or more. Adjusted Odds ratio (AOR), and 95% CIs are reported from multivariable modelling. An interaction term between gravidity and age was included in the multivariable model and the significance of the interaction term was tested using a Wald test. For the interaction between gravidity and age, stratum-specific AOR and 95% CIs are reported. The adequacy of the model was examined using a goodness of fit test that account for the survey design and survey weighting. Model specification was tested using the link test. Multi-collinearity between explanatory variables was assessed using variance inflation factor.

### Ethical considerations

Ethical approval was obtained from the University of the Witwatersrand Human Research Ethics Committee (Medical), and the nine provincial health research ethics committees. The study protocol was reviewed in accordance with the Centers for Disease Control and Prevention (CDC) human research protection procedures and was determined to be research, but CDC investigators did not interact with human subjects, or have access to identifiable data or specimens for research purposes.

## Results

**Fig 1:**
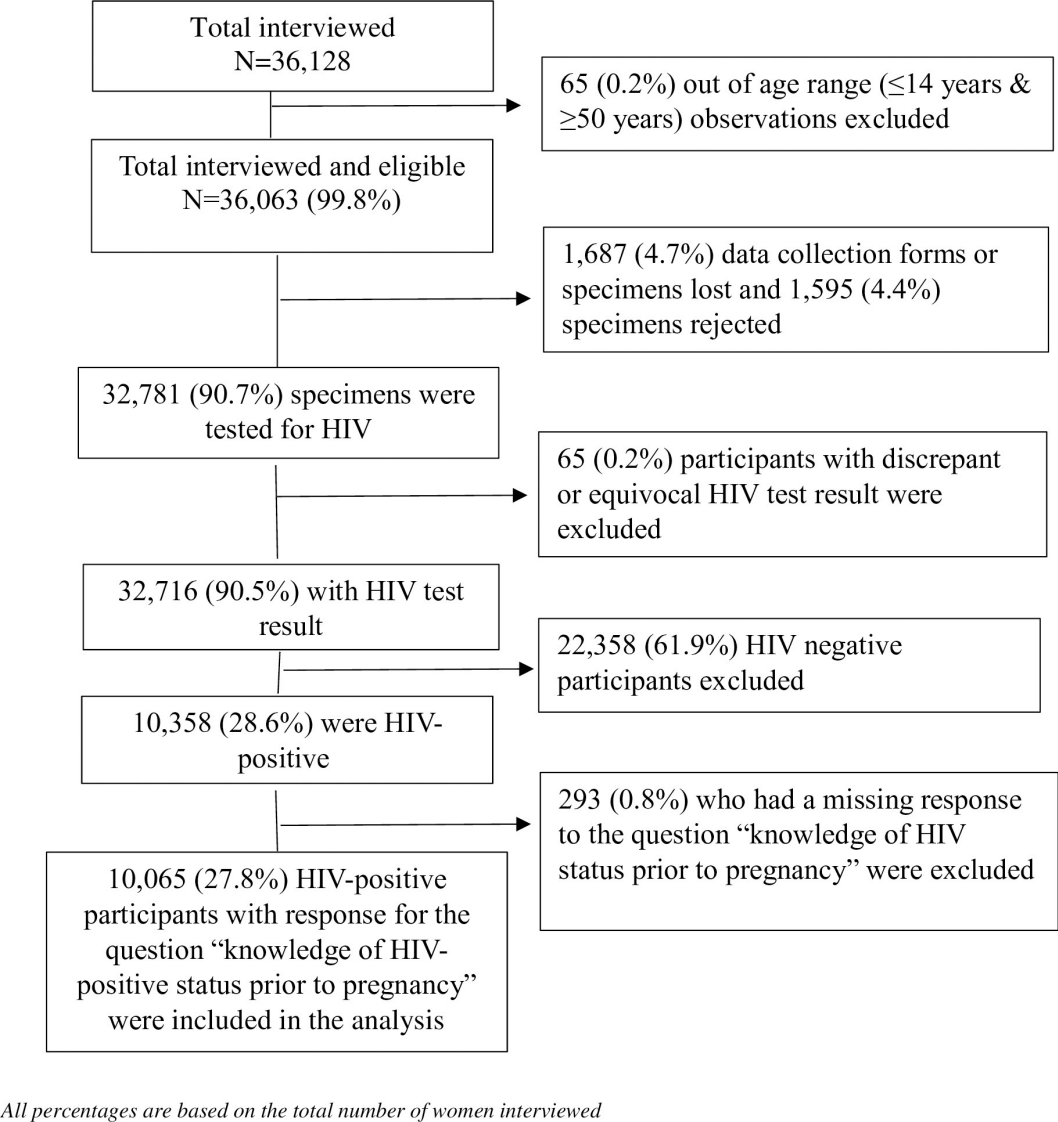
Flow chart of observations excluded from the analysis, national antenatal sentinel survey, 2017.

Thirty six thousand one-hundred twenty eight (36,128) participants were interviewed for the 2017 national survey (Fig 1). For this analysis, we have excluded 26,063 participants from the following categories: 1) outside of the age ranges (15–49 years) for inclusion (n = 65); 2) missing HIV test results or interview data (n = 1,687); 3) rejected blood specimens (n = 1,595) with 80% of specimen rejections due to haemolysis, 4) discrepant or equivocal results (n = 65), 5) HIV negative participants (n = 22,358), and 6) participants who had a missing response to the question “knowledge of HIV-positive status prior to the current pregnancy” (n = 293). In the final sample for analysis, 10,065 participants were included.

### Characteristics of survey participants

The median age of participants was 28 years (IQR: 24–33 years) (Table 1). The vast majority of participants were Black African (97.1%), single, that is, never married and not co-habiting (72.8%), and had attended at least secondary school (88.1%*)*. More than three-quarters (83.4%) of participants reported that the current pregnancy was not their first pregnancy and 79.7% had one or more children. Close to a third (32.1%) of participants were enrolled from public health care facilities in rural areas, 60.3% were from urban areas.

**Table 1:** Demographic characteristics of HIV-positive participants, in the 2017 antenatal HIV sentinel survey, SA.

Description	Sample distribution	(N = 10,065)
	Number	%
**Median (IQR) age in years**	28 (24–33)	
**Age in years**		
15–19	504	5.4
20–24	1,929	20.7
25–29	2,817	30.2
30–34	2,442	26.2
35–49	1,635	17.5
Missing	738	
**Marital status**		
Single	7,330	73.8
Married	1,514	15.2
Co-habiting	1,038	10.5
Divorced, separated and widowed	54	0.5
Missing	129	
**Population group**		
Black African	9,660	97.1
Other (Coloured, white, Asian)	285	2.9
Missing	120	
**Education**		
None	238	2.4
Primary	933	9.5
Secondary	7,721	78.8
Tertiary	914	9.3
Missing	259	
**Gravidity**		
Primigravida (1)	1,658	16.6
Multigravida (2+)	8,346	83.4
Missing	61	
**Number of live born children**		
0	2,028	20.3
1+	7,961	79.7
Missing	76	
**Geo-type**		
Urban	6,065	60.3
Rural	3,228	32.1
Peri-urban	772	7.7
**Province**		
Eastern Cape (EC)	1,330	13.2
Free State (FS)	877	8.7
Gauteng (GP)	1,515	15.1
KwaZulu- Natal (KZN)	3,325	33.0
Limpopo (LP)	575	5.7
Mpumalanga (MP)	1,051	10.4
North West (NW)	572	5.7
Northern Cape (NC)	266	2.6
Western Cape (WC)	554	5.5

Missing data not included when calculating percentages; unweighted data.

### Knowledge of HIV-positive status and ART initiation prior to pregnancy

**Fig 2:**
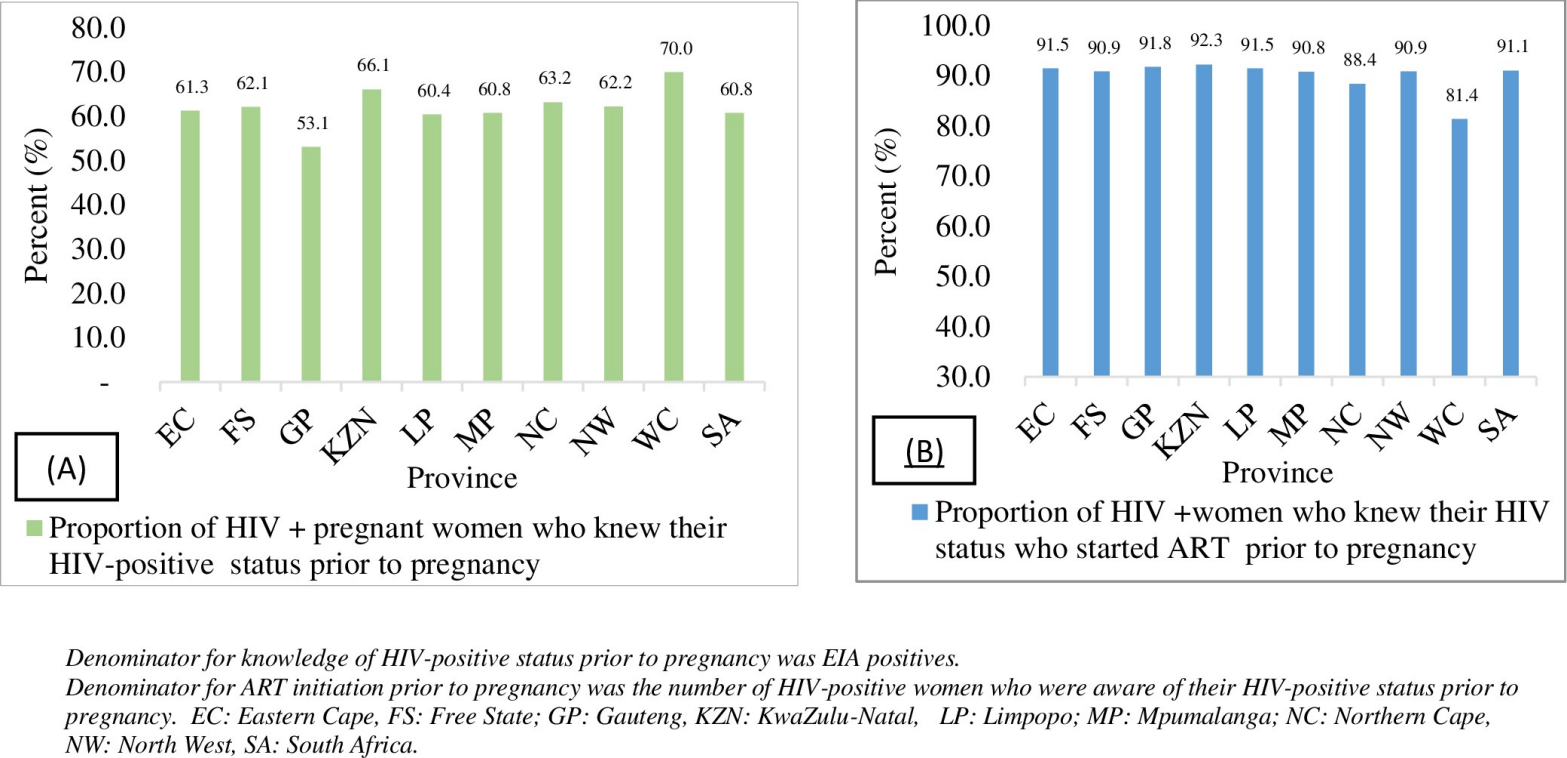
Knowledge of HIV-positive status (A) and ART initiation (B) prior to current pregnancy by province in the 2017 antenatal HIV sentinel survey, SA.

Of 10,065 HIV-positive participants, 60.8% (95% CI: 59.9%– 61.7%) knew their HIV-positive status prior to the current pregnancy. Among those who knew their HIV-positive status, 91.1% (95% CI: 90.4%–91.7%) were initiated on ART prior to the current pregnancy (Fig 2). This translated to 55.4% of all HIV-positive pregnant women who had started ART prior to the current pregnancy. Knowledge of HIV-positive status varied by province. The highest knowledge of HIV-positive status was in Western Cape (WC) (70.0%) and Kwazulu-Natal (KZN) (66.1%), whilst Gauteng (GP) had the lowest knowledge of HIV-positive status (53.1%). The lowest ART initiation was in WC (81.4%). All provinces except WC and Northern Cape (NC) had >90% coverage for ART initiation (the 2nd 90).

#### Knowledge of HIV-positive status and ART initiation by demographic characteristics

**Fig 3:**
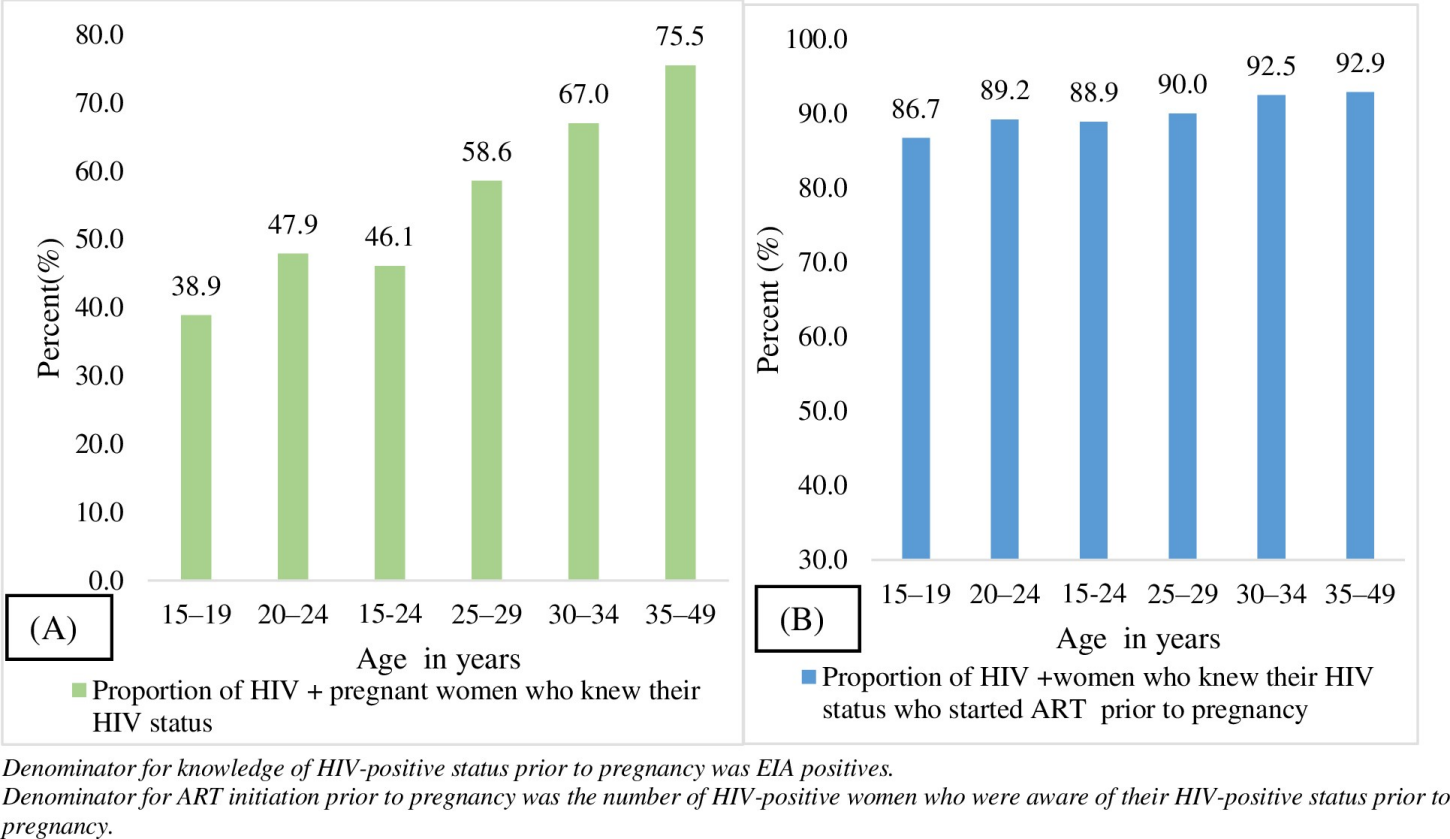
Knowledge of HIV-positive status (A) and ART initiation (B) prior to pregnancy by age group, in the 2017 antenatal HIV sentinel survey, SA.

Knowledge of HIV-positive status and ART initiation prior to the current pregnancy were higher (75.5% and 92.9% respectively) in the older age groups (35–49 years) compared with the younger age groups (38.9% and 86.7% respectively for 15–19 years; 47.9% and 89.2% respectively for 20–24 years) (P value: <0.01) (Fig 3). The 1^st^ and the 2^nd^ 90 prior to pregnancy among adolescent girls and young women (15–24 years) in this study is reported to be 46.1% and 88.9% respectively (Fig 3).

In a bivariable analysis, a significantly smaller proportion of primigravid women (40.5%) compared with multigravid women (64.7%) knew their HIV-positive status prior to first ANC visit (P value <0.01) (Table 2).

**Table 2:** knowledge of HIV-positive status and ART initiation prior to pregnancy by demographic characteristics, bi-variable analysis, antenatal HIV sentinel survey, 2017, SA.

	Women who knew their HIV-positive status prior to pregnancy visit				Women who started ART prior to pregnancy visit		
	HIV-positive (N)	Knew their HIV-positive status prior to pregnancy visit (n1)	weighted % (n1/N)	*P* value	Initiated ART prior to pregnancy visit (n2)	Weighted % (n2/n1)	*P* value
**Marital status**							
Single	7,330	4,408	59.2	0.4	3,953	90.8	0.1
Married	1,514	1,053	66.7		952	90.5	
Co-habiting	1,038	685	64		631	93.2	
Divorced and widowed	54	114	57.8		103	91.3	
**Education**							
None	238	166	69.8	0.06	147	91.7	0.3
Primary	933	616	66		568	93	
Secondary	7,721	4,770	61.8		4,293	91	
Tertiary	914	551	60.2		491	89.9	
**Gravidity**							
Primigravida (1)	1,658	688	40.5	<0.01	598	88.1	<0.01
Multigravida (2+)	8,346	5,540	64.7		5,012	91.4	
**Facility location**							
Urban	6,065	3,730	59.7	0.1	3,324	90.4	0.8
Rural	3,228	2,061	63.3		1,894	92.4	
Peri-urban	772	469	60.5		421	91.3	

Missing data excluded; ART–Antiretroviral therapy.

Univariable models for marital status, population group, education and facility location were adjusted for Age and gravidity. Population group was excluded from the logistic regression, as most (97.1%) participants were Black African.

In a multivariable analysis, after adjusting for education, and marital status, primigravidity and age were associated with knowledge of HIV-positive status (Table 3). Gravidity was an effect modifier in the association between age and knowledge of HIV-positive status (*P* value Wald test = 0.047). Among both primigravid and multigravid women, the odds of knowing HIV-positive status was lower among younger women compared with older women (35–49 years), with greater effects among primigravid women [AOR: 15–24 years: 0.2 (95% CI: 0.1–0.4), 25–29 years: 0.4 (95% CI: 0.2–0.6) and 30–34 years: 0.5 (95% CI: 0.2–0.9), using 35–49 years as a reference group] compared with multigravid women [AOR: 15–24 years: 0.4 (95% CI: 0.3–0.4), 25–29 years: 0.5 (95% CI: 0.4–0.6) and 30–34 years: 0.7 (95% CI: 0.6–0.8), using 35–49 years as a reference group].

**Table 3:** Demographic factors associated with knowledge of HIV-positive status prior to current pregnancy, in the 2017 antenatal HIV sentinel survey, SA.

	Unadjusted OR (95% CI)*	Adjusted OR (95% CI)**
**Age among primigravida**		
15–24 years	0.2 (0.1–0.4)	0.2 (0.1–0.4)
25–29 years	0.4 (0.2–0.7)	0.4 (0.2–0.6)
30–34 years	0.4 (0.2–0.8)	0.5 (0.2–0.9)
35–49 years	ref	ref
**Age among multigravida**		
15–24 years	0.4 (0.3–0.4)	0.4 (0.3–0.4)
25–29 years	0.5 (0.4–0.6)	0.5 (0.4–0.6)
30–34 years	0.7 (0.6–0.8)	0.7 (0.6–0.8)
35–49 years	ref	ref
**Gravidity among 15–24 years age group**		
Primigravida	0.5 (0.4–0.5)	0.5 (0.4–0.5)
Multigravida	ref	ref
**Gravidity among 25–29 years age group**		
Primigravida	0.5 (0.4–0.6)	0.6 (0.5–0.7)
Multigravida	ref	ref
**Gravidity among 30–34 years age group**		
Primigravida	0.5 (0.4–0.7)	0.6 (0.4–0.7)
Multigravida	ref	ref
**Gravidity among 35–49 years age group**		
Primigravida	0.8 (0.5–1.5)	0.8 (0.5–1.5)
Multigravida	ref	ref

Missing data excluded from logistic regression (n = 9,053 complete observations included in the multivariable logistic regression). Linktest p-value = 0.79; F-test (for goodness of fit) = 0.99; P-value from Wald test (for interaction term)- 0.047. Adjusted for marital status, and education.

* bi-variable model.

** multivariable model.

Primigravidity was associated with lower knowledge of HIV-positive status in all age groups except among the age group 35–49 years. This could be because most (97.6%) of the participants in the age group 35–49 years were multigravida.

Due to multi-collinearity effect between parity and gravidity, parity was not included in the multivariable logistic model, but both variables were influential in a multivariable model. The Hosmer Lemeshow statistics for goodness of fit (*P* value = 0.99) showed the model fits the data well. The link test performed to assess model specification showed the model is correctly specified (*P* value = 0.79).

## Discussion

In this nationally representative study of women seen at public ANC facilities in 2017, nearly 40% of HIV-positive women were unaware of their HIV status at the time of their first ANC visit. This increased to about 60% among adolescent girls (15–19 years) and primigravid women. For this segment of the South African population, the country is far short of the UNAIDS target, for 90% of people to know their HIV status.

Higher knowledge of HIV-positive status and ART initiation prior to pregnancy was observed among multigravid women and older women (35–49 years) compared with adolescent girls and primigravid women. This is expected as testing at ANC is almost universal which would account for women with prior pregnancy history having been tested previously. With increasing age, the chances of having had an HIV test increases as there is a much greater chance of having been exposed to the offer of a test. Conversely, access and uptake of HIV testing among adolescent girls and young women (15–24 years) is reported to be low in SA [14]. Despite high HIV incidence rate among adolescent girls and young women in SA, in the 2016 demographic and health survey (DHS), just over one-third (38.4%) of adolescent girls (15–19 years) and 52.5% of young women (15–24 years) reported testing for HIV within the last 12 months preceding the survey [15, 16]. In light of the high incidence rate of HIV among adolescent girls and young women, the gap in HIV testing uptake among this age group poses a significant challenge in the effort to achieve epidemic control in SA. Innovative strategies are needed to address barriers to HIV testing among adolescent girls and young women. Such innovative strategies could include large-scale implementation of peer driven models, distribution of HIV self-screening kits in home and community settings and ensuring reproductive health education is integrated in schools curriculum [17–19].

The result from this study was comparable with data from the routine PMTCT programme. According to routine PMTCT data, between September 2016 and August 2017, 52.4% of pregnant women were initiated on ART prior to pregnancy [20], which is comparable with the 55.4% pre-conception ART coverage we report in this survey from data collected in the months of October and November, 2017. Based on routine programme data, in the year prior to the introduction of the UTT strategy (September 2015–August 2016), 42.6% of pregnant women were initiated on ART prior to pregnancy, indicating a 23% increase in ART coverage (from 42.6% to 52.4%) within one-year of implementation of the UTT strategy [20]. While the implementation of the UTT strategy needs continued strengthening to achieve the 90–90–90 targets, the observed progress in the number of HIV-positive women who initiate ART prior to pregnancy is encouraging as this will help achieve the elimination of mother-to-child transmission target as well as increase ART coverage among pregnant women and potentially other populations such as partners of these women.

This study provided the first national data for the 1^st^ and 2^nd^ 90 UNAIDS targets among adolescent girls and young women in South Africa. Previous studies reported the 90-90-90 achievement geographically and by gender, whereas no study could be found that report at national level the progress in the 1^st^ and 2^nd^ 90 among adolescent girls and young women.

The first 90 (60.8%) reported in this survey for reproductive age (15–49 years) women was lower than the 1st 90 reported for the same age group in the 2017 South African household survey (88.9%) and in the UNAIDS report (92%) [16, 21]. Methodological differences between these studies and the antenatal survey may partially explain the difference. The antenatal survey used self-reported HIV status data extracted from the medical record to determine participants’ knowledge of HIV status. These data may be subjected to self-reporting bias as women who know their HIV-positive status but are not linked to treatment may under report knowledge of HIV status prior to pregnancy, and even if this information was reported accurately it may not always be recorded in the patient file. In the South African household survey, in addition to self-reported HIV status, HIV biomarkers (ARV drug level and viral load) were used to determine participants’ knowledge of HIV-positive status, which is likely to improve the accuracy of the estimate [22]. The difference could also be explained by the risk profile of pregnant women. Pregnant women by definition are women who recently had unprotected sex. Unprotected sex increases the risk of HIV exposure if the couples had not tested for HIV, were not virally suppressed, or safer options for conception were not used. Studies report high-risk behaviours (such as frequent unprotected sex) lead to both new HIV infection and unplanned pregnancy [23, 24].

According to this survey, the UNAIDS target for the second 90 (ART coverage) is achieved at national level and in seven of the nine provinces. However, this result needs to be interpreted with caution as under reporting of the denominator “knowledge of HIV-positive status” may result in overestimation of the second 90.

In a sensitivity analysis, knowledge of HIV-positive status and ART coverage did not vary between first ANC visit attendees and follow-up ANC visit attendees, indicating new sero-conversion during pregnancy may not explain the low knowledge of HIV-positive status prior to pregnancy. To assess bias associated with missing data, we performed sensitivity analysis by including HIV positive participants with interview data but missing laboratory data–the coverage estimate for both the 1^st^ and the 2^nd^ 90 stayed the same after including in the analysis HIV positive participants with interview data but missing laboratory data. Including in the denominator, HIV-positive participants with laboratory data but missing interview data, reduce the 1^st^ 90 estimate by a small percentage (by 1%). Access to ANC (99%) and attendance of ANC is high (>94%) in SA [15]; however, the findings of this survey do not apply to women who use antenatal services in the private health sector as the study was limited to public sector clinics. The results of this study also do not apply to small facilities (facilities with <20 first-time ANC clients per month), but majority (85%) of ANC facilities in SA serve >20 first-time ANC clients per month.

The use of two 4^th^ generation EIA tests with no confirmatory test (e.g. Western blot) may result in a slightly elevated number of false positive results, however, in our study there was a high (>95%) concordance between the survey EIA test result and the rapid test result performed in the ANC clinic, suggesting false positive EIA results may not have a significant effect on the findings of the study. Participants with missing age data were not excluded from crude analysis as rapid review of the participants’ medical record confirmed that participants with missing age data were eligible (within the age range) for inclusion in the survey but due to clerical errors their age was not captured.

## Conclusion

This study provides the first national level data for tracking progress towards the 1^st^ and 2^nd^ 90 among sexually active adolescent girls and young women in South Africa. Despite other reports indicating the achievement of the 1st 90 target among women in SA [21], our survey shows knowledge of HIV-positive status prior to pregnancy was low among women, especially amongst adolescent girls, 15–19 years, and 20–24 years old women. The low knowledge of HIV status prior to pregnancy could be due to both low uptake of HIV testing and high-risk behaviours prior to pregnancy that may lead to both new HIV acquisition and unplanned pregnancy. Increasing HIV testing coverage among adolescent girls and young women is essential as the available evidence indicates HIV testing coverage is low in this age group. We recommend increasing access to youth-friendly HIV testing and reproductive health and health education services at various levels, including schools, communities, and facilities. In addition, achieving the 1st 90 target without strengthening prevention efforts could be difficult if individuals after having tested for HIV continue to practise risky behaviours. Therefore, there is a need to strengthen prevention efforts, including increasing awareness and access to pre-exposure prophylaxis (PrEP) and post-exposure prophylaxis (PEP) services for high-risk population groups, such as adolescent girls and young women. It is also beneficial to integrate family planning services into HIV testing and treatment services and increase awareness about options for safer conception for women who want to be pregnant while in a discordant relationship.
